# Knowledge, Attitudes, and Practices to Periodontal Health of the Northeast Chinese Public: Cross-Sectional Study

**DOI:** 10.2196/72069

**Published:** 2025-08-22

**Authors:** Chengde Jin, Ming Chi, Chunxiao Li, Yushan Li, Wanting Wang, Yushi Zhang, Qiao Liu

**Affiliations:** 1 China Medical University, Shenyang City, China; 2 Huludao Central Hospital, Huludao, Liaoning Province, 125000, China, 86 13372900271

**Keywords:** KAP, sociodemographic factors, gingival bleeding, Knowledge, Attitudes, and Practices, periodontal health awareness

## Abstract

**Background:**

Periodontitis affects approximately 50% of adults in China and is a leading cause of tooth loss in this population. However, there is a notable paucity of research on knowledge, attitudes, and practices (KAP) related to periodontitis among patients in Northeast China.

**Objective:**

This study aimed to investigate the KAP regarding periodontitis among populations in Northeast China, focusing on 5 demographic factors: gender, age, income, education level, and region.

**Methods:**

A cross-sectional survey was conducted by convenience sampling over a period of 1 week. A structured questionnaire was used to collect detailed responses on periodontitis-related KAP. Descriptive statistics (means, SDs, frequencies, and percentages) were used. Normality was assessed by Shapiro-Wilk tests. Nonparametric Kruskal-Wallis and multivariate regression analyses were conducted to examine demographic influences and interactions on periodontal KAP scores. Statistical significance was defined at *P*<.05.

**Results:**

A total of 619 questionnaires were distributed, resulting in 562 valid responses comprising 242 (43.06%) males and 320 (56.94%) females, with a mean participant age of 41.27 (95% CI 37.4‐45.1) years. The overall awareness of periodontal disease was relatively low in Northeast China, with the mean KAP scores being 3.88/8, 5.28/7, and 5.19/11. Age and educational level were both significantly associated with individuals’ KAP regarding periodontitis (*P*<.05), whereas gender showed a significant association with knowledge only (*P*<.05). Regional and income-related differences were generally significant, with only a variable showing marginal effects (*P*=.05). Multiple regression analysis indicated that both knowledge and attitude scores tend to increase with age. The increase in knowledge was most pronounced in the age group of 41‐50 (Nagelkerke Pseudo Coefficient of Determination (Nagelkerke R^2^)=1.58, 95% CI 0.83‐2.33; *P*<.01) years. Attitude scores exhibited a more consistent upward trend across all age groups. In contrast, practice scores declined with age. In terms of interactions, young females exhibited significantly higher awareness than males, whereas no significant gender differences were observed among older populations. In addition, higher education levels and economic status were strongly associated with improved awareness. Notably, the presence of gingival bleeding significantly enhanced public awareness of periodontitis, especially knowledge score (Nagelkerke R^2^ =1.07, 95% CI 0.69‐1.44; *P*<.01).

**Conclusions:**

This study provides a comprehensive understanding of the KAP regarding periodontitis among populations in Northeast China. The findings offer valuable insights for the formulation of targeted policies and underscore the importance of improving periodontal KAP in the region.

## Introduction

Periodontitis is the sixth most prevalent chronic noncommunicable disease worldwide [1], characterized by inflammation and destruction of the 4 periodontal supporting tissues [2]. This condition can cause tooth loosening, displacement, and eventual loss, resulting in impaired chewing and speaking abilities [3]. Furthermore, periodontitis is implicated in the development of various systemic conditions, including cardiovascular disease, type 2 diabetes, rheumatoid arthritis, and inflammatory bowel disease [4]. These complications significantly diminish patients’ quality of life.

Globally, periodontitis affects approximately 12.5% of the population and ranks as the second most common oral disease after dental caries in permanent teeth, impairing oral health for over 1 billion people worldwide [5,6]. A recent meta-analysis estimated that about 62% of dentate adults have periodontitis, with approximately 23.6% classified as severe cases, marking a significant increase compared to prevalence rates from 1990 to 2010 [7]. The situation in China is similarly concerning, as gingival inflammation affects as many as 61.0% of adolescents aged 14 years, and over half of the individuals aged 35 years and older have periodontal disease, which notably deteriorates with increasing age [8,9]. Despite the widespread prevalence of periodontal disease, it remains insufficiently addressed due to its silent progression and low public awareness, with patients typically seeking treatment only at advanced stages [2,10,11]. Consequently, this delay significantly exacerbates the burden associated with periodontitis.

The primary risk factor for periodontitis is the accumulation of microbial plaque within the oral cavity [12]; thus, maintaining good oral hygiene is essential for the effective prevention of periodontal disease. Effective plaque control through proper brushing techniques, flossing or interdental cleaning, and using fluoride-containing toothpaste and mouthwash is critical to maintaining oral hygiene [13]. Although preventive measures appear straightforward theoretically, their implementation at both individual and public health levels often presents significant challenges [14]. Studies indicate that the high prevalence of periodontal disease is strongly associated with inadequate public awareness and understanding of the disease [15]. Therefore, investigating public awareness regarding periodontitis and formulating corresponding policies are crucial steps toward reducing its prevalence and burden.

Knowledge, Attitudes, and Practices (KAP) surveys represent a structured research method designed to assess individuals’ understanding, perceptions, and behaviors related to specific health conditions or preventive practices. These studies aim to identify obstacles hindering the adoption of desirable health behaviors, highlight specific health concerns within target and broader populations, and inform the implementation of targeted oral health education policies based on the findings, ultimately enhancing community health awareness and easing public health burdens [16-18].

Understanding the KAP of the population regarding periodontitis is vital for developing targeted oral health education policies tailored to different demographic groups. It also facilitates the creation of referral pathways for patients with periodontal disease and comorbid medical conditions, thereby supporting the integration and transition of care. These efforts are essential for maintaining oral health in older adults and achieving the “80‐20” goal, which aims for individuals aged 80 years to retain at least 20 functional teeth. Although nationwide studies have investigated periodontal knowledge and behaviors in China [19], or have been limited to surveys of individuals with periodontitis [20], no research has specifically addressed the northeastern population. Crucially, prior studies have neither systematically examined how sociodemographic variables interact to shape periodontal KAP in this region nor have they considered the role of gingival bleeding as a contributing factor. This study addresses this gap using a representative sample from northeastern China to evaluate the multivariate and combined effects of 5 key demographic factors, such as gender, age, education, income, and geographic classification, on periodontal KAP. It identifies high-priority subpopulations for policy intervention and offers insights potentially generalizable to other regions with similar demographic profiles.

## Method

### Questionnaire

The questionnaire consisted of 23 items divided into 5 sections, with all questions carefully designed to avoid leading or ambiguous language. The first section collected basic demographic information. The second section assessed participants’ knowledge of periodontitis through 8 multiple-response questions. The third section evaluated attitudes toward periodontitis with 3 questions. The fourth section measured participants’ practices with 5 questions, focusing on daily oral care and professional dental care. The fifth section included a question on gingival bleeding, which served as an indicator of potential periodontal disease. As supplementary information to the questionnaire, the title clearly outlined the study objectives, emphasized voluntary participation, detailed privacy protection measures, and informed participants that the collected data would serve as raw data for publication. A statement, “I have read and understood the information provided in the title and voluntarily agree to participate in this survey,”was included as a question item, with “Agree” or “Disagree” as response options. In addition, if participants selected an age between 12 and 17 years, an additional item querying guardian consent (“Yes” or “No”) was presented.

### Sample Method and Recruitment

A cross-sectional survey using a convenience sampling method was conducted between December 31, 2024 and January 7, 2025, to evaluate KAP regarding periodontitis in Northeast China. Prior to questionnaire distribution, a pilot study involving these 42 individuals was conducted to ensure clarity and comprehensibility of all questions.

This cross-sectional study was conducted across 3 provinces in Northeast China (Liaoning, Jilin, and Heilongjiang), encompassing both economically higher-income and lower-income regions to ensure demographic and socioeconomic diversity. From each province, 1 economically developed and 1 underdeveloped city were randomly selected, resulting in 6 cities. Within each city, 1 urban district and 1 rural township were randomly chosen, yielding a total of 12 distinct regions for participant recruitment.

Recruitment was conducted via a community-based approach. Local community health centers, secondary schools, and residential committees in the selected districts and townships collaborated in disseminating the questionnaire link to potential participants through WeChat (Tencent; a widely used communication platform in China). Volunteers from local health institutions, including public health nurses and dental practitioners, assisted in explaining the survey and encouraging participation. They also ensured that participants understood the purpose of the study and were available to address any questions. The online questionnaire was distributed via the Wenjuanxing (Changsha Ranxing Information Technology Co Ltd) platform, and participation was entirely voluntary.

### Evaluation Criterion

The scoring criteria were as follows: Questionnaire items were divided into 3 categories. For single-choice questions with 1 correct answer, responses were scored as either correct (1) or incorrect (0). Likert-scale items were scored on a scale from −2 to 2. Items assessing daily behaviors were scored from 1 to 3 based on the extent to which responses aligned with established behavioral standards.

The criteria for inclusion and exclusion were as follows: responses were excluded if participants did not explicitly consent to the informed consent and privacy policy. For participants aged between 12 and 17 years, additional consent from a guardian was required. Questionnaire responses relating to KAP that demonstrated clear irregularities, such as selecting identical answers for all items, were also excluded. As the questionnaire included prompts for unanswered questions, there were no missing values. All remaining data were retained for subsequent analyses.

### Reliability Analysis and Validity Assessment

To assess the internal consistency of the questionnaire, Cronbach α was calculated for each KAP dimension. The results were as follows: Knowledge (α=0.437), Attitude (α=0.417), and Practice (α=0.319). These values suggest moderate to low internal consistency, which may be attributed to the multifaceted nature of the questionnaire. Since the study primarily focuses on descriptive statistical analysis and regression modeling rather than scale validation, no further item reduction or confirmatory factor analysis was performed. To evaluate the suitability of the dataset for correlation and regression analyses, Bartlett test for sphericity was conducted. The test yielded a significant result (*χ*²_153_=1159.3; *P*<.001), indicating sufficient correlation among variables.

### Statistical Methods

All statistical analyses were performed using IBM SPSS Statistics 27.0 (IBM Corp), RStudio (Posit, PBC), and WPS Office (Kingsoft Office Software). Data were first checked for normality using the Shapiro-Wilk and D’Agostino-Pearson tests, which confirmed that the KAP scores were non-normally distributed (*P*<.05). Accordingly, nonparametric statistical methods were used for comparisons. Given the categorical nature of sociodemographic variables (eg, gender, age, region, income, and education level) and the continuous nature of KAP scores, Kruskal-Wallis tests were used to compare multiple groups. For pairwise comparisons, the Dunn test with Benjamini-Hochberg correction was used to control the false discovery rate. In cases involving multiple hypothesis testing (eg, age groups with 7 categories), Holm correction was applied to maintain the conservativeness of the results. Statistical significance was set at *P*<.05 for all analyses.

For multivariate analysis, multiple regression models were used to examine the effects of sociodemographic factors on KAP scores. Interaction effects between variables (eg, age×gender and income×education) were explored to assess their impact on knowledge, attitude, and practice scores. Data visualization was performed using grouped bar charts, line graphs, and regression coefficient plots to provide an intuitive representation of differences across demographic groups.

### Ethical Considerations

The study received ethical approval from the Medical Ethics Committee of the Affiliated Stomatological Hospital, China Medical University (2025002). Prior to participation, the questionnaire clearly outlined the study’s objectives and content and assured participants that all data would be used solely for research purposes with strict confidentiality. Participants were informed of their right to withdraw from the study at any time without penalty or consequence. All data were collected with informed consent from participants, including additional parental consent for minors aged 12‐17 years. All data collected in this study were anonymized and stored securely. No personally identifiable information was retained or analyzed. Participants did not receive any monetary or material compensation for their involvement in this research. No images of participants are included in the paper or supplementary materials. Therefore, there is no risk of participant identification. Otherwise, respondents were required to read an information statement embedded in the questionnaire title explaining the purpose, confidentiality, and voluntariness of the study. Informed consent was obtained digitally before submission. For respondents aged 12–17 years, a mandatory additional item was included to verify guardian consent before their data were considered valid.

## Results

### Basic Information

A total of 619 questionnaires were distributed. Among these, 562 were valid responses, with 242 (43.06%) from males and 320 (56.94%) from females. The average age of the participants was recorded as 41.27 (95% CI 37.4-45.1).

### Knowledge

**Figure 1.**
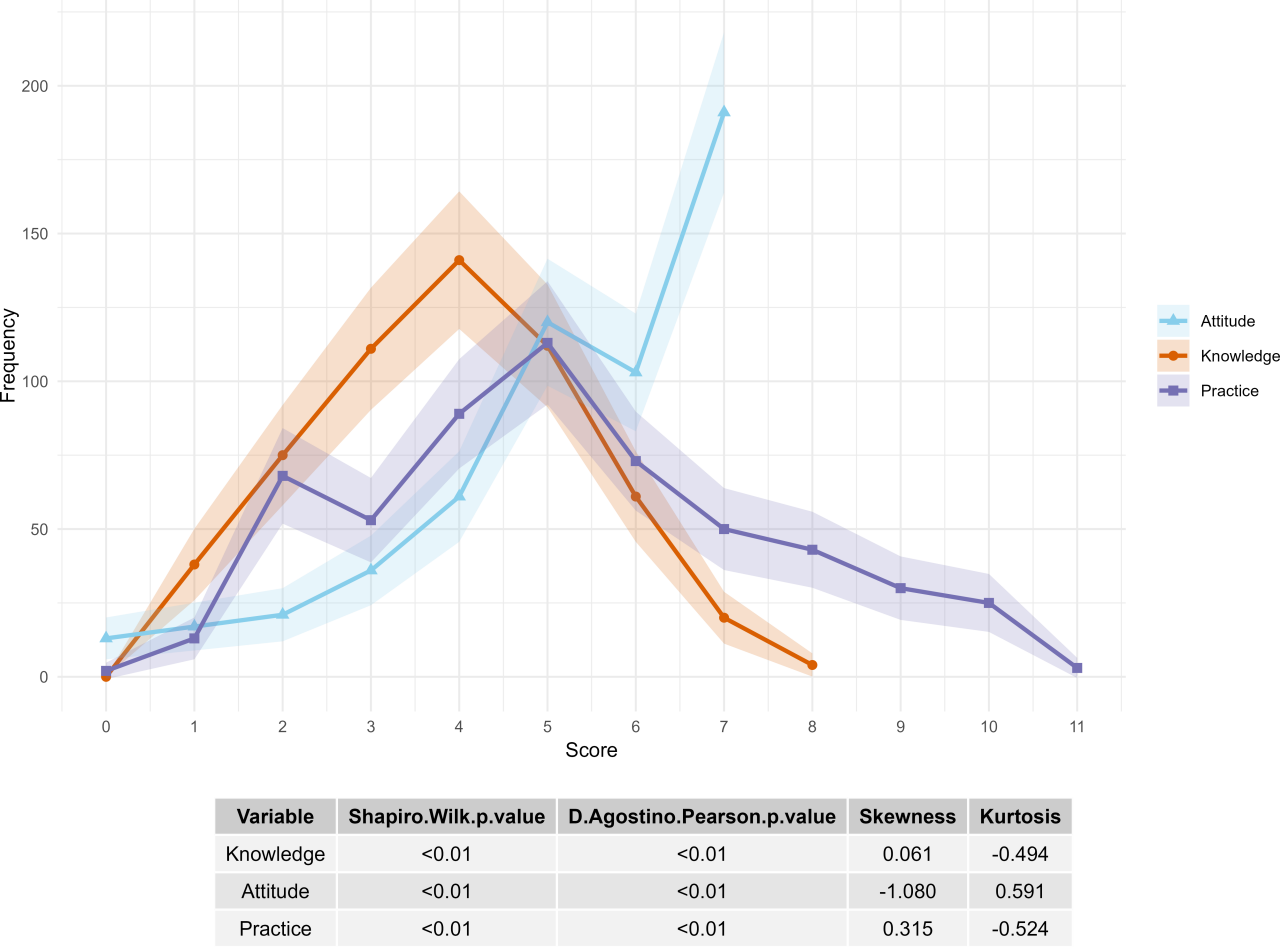
Frequency distribution of scores across Knowledge, Attitude, and Practice categories on periodontitis (Northeast China, Dec 31, 2024 – Jan 7, 2025). This line chart illustrates the frequency distribution of scores for Knowledge (orange), Attitude (blue), and Practice (purple) regarding periodontitis prevention and awareness. The x-axis represents the score values while the y-axis indicates frequency, measured as the number of participants who achieved each score. The maximum possible scores vary by category: Knowledge (0‐8), Attitude (0‐7), and Practice (0‐11). Shaded areas represent 95% CIs, reflecting variability in score distributions across the population. Statistical tests (Shapiro-Wilk and D’Agostino-Pearson) showed significant deviations from normality in all 3 domains (*P*<.01).

The average knowledge score of participants regarding periodontitis was 3.88 out of 8 (Table 1). Normality tests using the Shapiro-Wilk and D’Agostino-Pearson tests (both *P*<.05), combined with a skewness of 0.06 and kurtosis of −0.49 (Figure 1), indicated that the knowledge scores were primarily concentrated at the lower end, with fewer extreme low and high values. This distribution reflects generally poor awareness of periodontitis among the population.

**Table 1.** Characteristics of the participants and knowledge, attitude, and practice scores and proportion of gingival bleeding on periodontitis among participants in Northeast China (Dec 31, 2024–Jan 7, 2025).

Characteristics	Value (n)	Knowledge score			Attitude score			Practice score			Gingival bleeding rate (%)
		Mean (SD; 95% CI)		*P* value	Mean (SD; 95% CI)		*P* value	Mean (SD; 95% CI)		*P* value	
All	562	3.88 (1.55; 3.76-4.01)		<.01^b^	5.28 (1.80; 5.13-5.43)		<.01^b^	5.19 (2.32; 5.00-5.38)		.14	51.42
Sex				.03^c^			.23			.73	
Man	242	3.72 (1.44; 3.54-3.90)			5.19 (1.81; 4.97-5.42)			5.14 (2.26; 4.85-5.42)			53.31
Women	320	4.01 (1.62; 3.83-4.18)			5.34 (1.79; 5.14-5.54)			5.22 (2.37; 4.96-5.48)			50.00
Age (years)				.02^c^			.03^c^			.04^c^	
12-17	19	3.26 (1.91; 2.34-4.18)			3.68 (2.16; 2.64-4.73)			6.21 (2.35; 5.08-7.34)			57.89
18-25	89	4.19 (1.43; 3.89-4.49)			5.69 (1.25; 5.42-5.95)			4.67 (2.53; 4.14-5.21)			51.69
26-30	65	3.14 (1.29; 2.82-3.46)			4.26 (2.25; 3.7-4.82)			5.77 (2.04; 5.26-6.28)			50.77
31-40	167	3.78 (1.46; 3.56-4)			5.45 (1.54; 5.21-5.68)			5.49 (2.18; 5.15-5.82)			56.29
41-50	99	4.63 (1.58; 4.31-4.94)			6.08 (1.25; 5.83-6.33)			4.8 (2.38; 4.32-5.27)			46.46
51-60	53	3.66 (1.39; 3.28-4.04)			5.79 (1.67; 5.33-6.25)			4.72 (2.13; 4.13-5.31)			32.08
more than 60	70	3.73 (1.63; 3.34-4.12)			4.2 (2.03; 3.72-4.68)			5.2 (2.43; 4.62-5.78)			60.00
Region				.05			.02^c^			.03^c^	
Rural areas	259	4.36 (1.42; 4.19-4.53)			5.71 (1.78; 5.49-5.92)			4.98 (2.44; 4.68-5.28)			47.76
Small cities	119	3.76 (1.46; 3.49-4.02)			5.26 (1.42; 5-5.52)			5.97 (2.13; 5.58-6.35)			52.14
Midsized cities	117	3.17 (1.63; 2.87-3.47)			4.52 (1.79; 4.19-4.85)			5.14 (1.91; 4.79-5.49)			63.87
Metropolitan areas	67	3.52 (1.47; 3.16-3.88)			4.97 (2.01; 4.48-5.46)			4.67 (2.57; 4.05-5.3)			46.33
Education				.03^c^			.04^c^			.01^b^	
High school or below	143	3.88 (1.47; 3.64-4.12)			5.12 (1.76; 4.83-5.41)			5.25 (2.28; 4.88-5.63)			60.84
Associate	128	3.55 (1.61; 3.27-3.84)			5.55 (1.46; 5.3-5.81)			5.57 (2.21; 5.18-5.96)			44.53
Bachelor	217	4.07 (1.49; 3.87-4.27)			5.52 (1.81; 5.27-5.76)			5.06 (2.22; 4.77-5.36)			53.46
Master or above	74	3.92 (1.71; 3.52-4.32)			4.41 (2.09; 3.92-4.89)			4.74 (2.77; 4.10-5.39)			39.19
Annual income^a^				.04^c^			.01^b^			.05	
Low	62	4.31 (1.30; 3.98-4.64)			4.98 (1.49; 4.61-5.36)			4.45 (1.79; 4.00-4.91)			40.32
Low-middle	201	3.61 (1.65; 3.38-3.84)			5.35 (1.57; 5.14-5.57)			5.32 (2.43; 4.98-5.66			55.22
Middle	194	3.85 (1.33; 3.66-4.04)			5.47 (1.83; 5.22-5.73)			5.48 (2.28; 5.16-5.81)			55.67
High-middle	68	4.09 (1.58; 3.71-4.47)			5.21 (1.96; 4.73-5.68)			4.76 (2.18; 4.24-5.29)			51.47
High	37	4.49 (2.02; 3.81-5.16)			4.46 (2.63; 3.58-5.34)			4.89 (2.71; 3.99-5.79)			27.03

a Annual income five groups: less than 30,000 (US $4225), 30,000-80,000 (US $4225-US $11,268), 80,000-150,000 (US $11,268-US $21,127), 150,000-300,000 (US $21,127-US $42,254), and more than 300,000 ( more than US $42,254).

b **P<0.01.

c *P<0.05.

**Figure 2.**
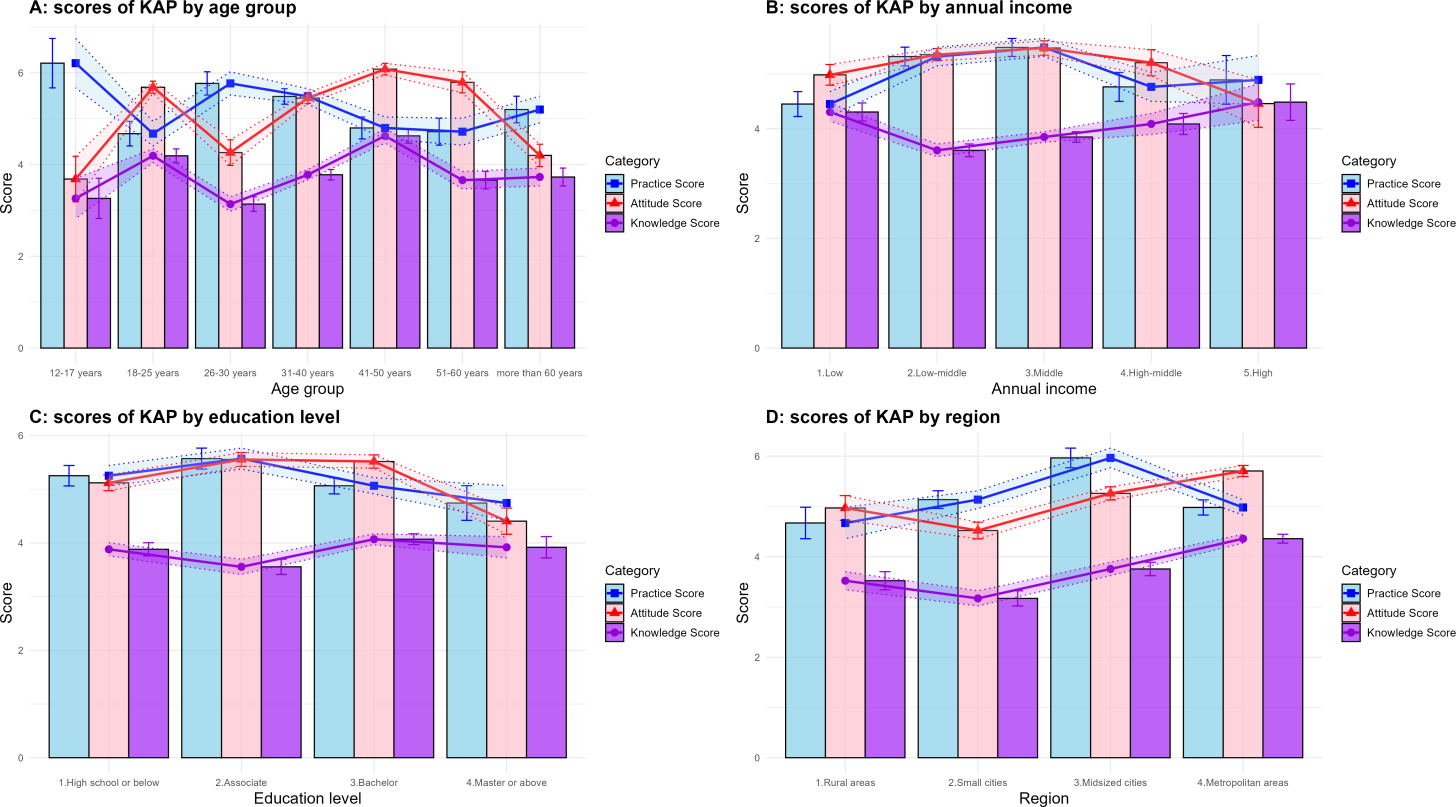
Mean scores of Knowledge, Attitude, and Practice (KAP) on Periodontitis Across Demographic Groups in Northeast China (Dec 31, 2024 – Jan 7, 2025). This figure shows the mean scores of Knowledge (purple line), Attitude (red line), and Practice (blue line) related to periodontitis. Error bars represent SEs of the mean. Statistical significance of differences between groups can be inferred from nonoverlapping error bars.

In terms of demographic characteristics, gender, age, region, education level, and annual income all had significant effects on knowledge scores (*P*<.05). Group analysis revealed that females had a mean knowledge score of 4.01 (SD 1.62; 95% CI 3.83‐4.18), significantly higher than males, whose mean score was 3.72 (SD 1.44; 95% CI 3.54‐3.90). Age-specific analysis showed 2 peaks in knowledge scores: 4.19 (SD 1.43; 95% CI 3.89‐4.49) in the age group of 18‐25 years and 4.63 (SD 1.58; 95% CI 4.31‐4.94) in the age group of 41‐50 years. Participants with higher annual incomes demonstrated higher knowledge scores. However, unexpectedly high scores were also observed in low-income regions, which may be attributed to variations in regional development levels. Education level was positively correlated with knowledge scores (*P*<.05), with higher education levels associated with better scores (Table 1, Figure 2). However, the differences in scores between educational levels were relatively small, with a maximum intergroup difference of only 0.11.

### Attitude

The overall attitude scores were relatively high, with a mean score of 5.28 out of 7 (Table 1). The skewness was −1.08, and the kurtosis was 0.59 (Figure 1), indicating a generally positive attitude toward periodontitis among the population.

**Figure 3.**
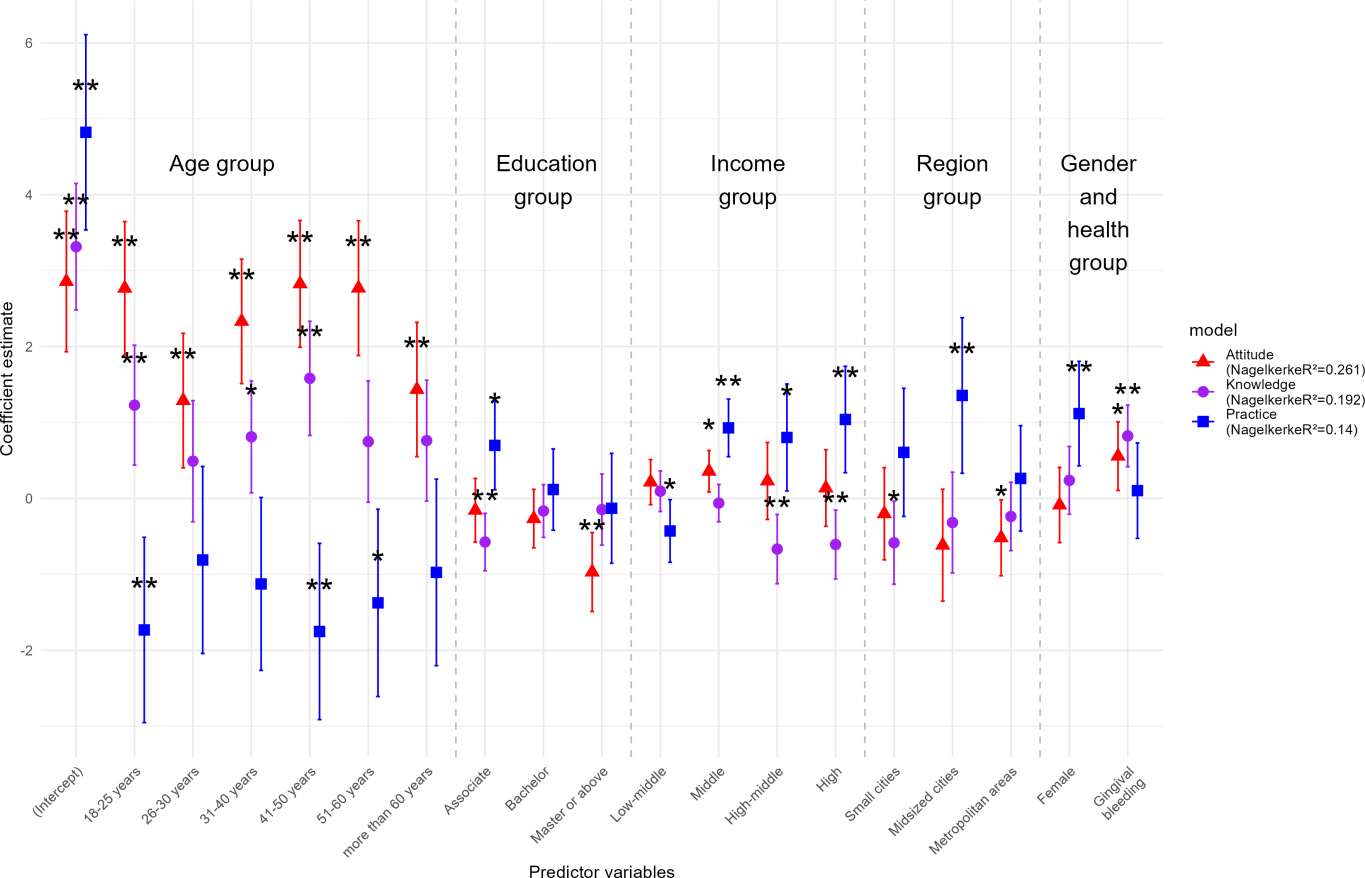
Regression coefficients for the Knowledge, Attitude, and Practice scores across various predictor variables (unstandardized estimates). Error bars represent the 95% CIs. **P<.05 and **P<.01.

Apart from gender (*P*=.23), all other sociodemographic characteristics showed significant differences in attitude scores. Group analysis revealed that attitude scores across different age groups and regions followed a trend similar to the variations observed in knowledge scores. However, among income groups, a significant decline in attitude scores was observed in the high-income population. This decline may be related to the lower prevalence of gingival bleeding in the high-income group (27.03%) compared to other groups (40.32%‐55.67%). Further analysis demonstrated that participants with gingival bleeding had significantly higher attitude scores than those without gingival bleeding (*P*<.05). In addition, attitude scores across different educational levels were also influenced by the presence of gingival bleeding (Table 1, Figure 3).

### Practice

Practice scores showed a peak around 5 but displayed a long-tailed distribution on the right, indicating substantial variability in the data (Figure 1). Despite the right-skewed trend, the mean practice score was 5.19 out of 11 (Table 1). This suggests that, while the overall practice level was relatively low, a small proportion of individuals achieved higher scores.

Significant differences in practice scores were observed across all demographic groups except gender (*P*<.05). However, univariate analysis revealed a significant correlation between gender and practice scores, with females outperforming males (*P*<.01). Age group analysis indicated a peak in practice scores among participants aged 12‐17 years, with a range of 5.08‐7.34. Regionally, participants from medium-sized cities exhibited the highest practice scores, which may be linked to the higher prevalence of gingival bleeding in this group (63.87%) compared to others (46.33%‐52.14%). However, further analysis revealed that participants with gingival bleeding had no significantly higher practice scores than those without gingival bleeding (*P*>.05) (Table 1, Figure 3).

### Nagelkerke Pseudo Coefficient of Determination

Multivariate regression analysis revealed that gender had no significant effect on KAP scores, while age was significantly associated with all dimensions of KAP. Knowledge and attitude scores were positively correlated with age, whereas practice scores showed a negative correlation. However, the influence of age diminished with increasing age, indicating that in older populations, age had minimal impact on scores. Specifically, knowledge scores significantly increased in all age groups compared to the age group of 12‐17 years, except for the age group of 26‐30 years. The age group of 41‐50 years showed the highest increase (Nagelkerke R^2^=1.58, 95% CI 0.83‐2.33; *P*<.01). Attitude scores exhibited a trend similar to knowledge score,s while practice scores were negatively correlated with age, with lower significance observed in the age groups of 26‐30 years and more than 60 years. Annual income had a negative correlation with knowledge scores, with high-income groups scoring significantly lower than low-income groups. No significant differences were observed in attitude scores among income groups. However, medium- and high-income groups exhibited a significant positive influence on practice scores. The effects of education level and region on KAP scores varied across specific populations (Figure 3).

A significant interaction between gender and age was observed in practice behaviors, with women more than 60 years scoring significantly higher than their male counterparts (interaction coefficient=3.70*; P*<.01), suggesting that older women tend to engage more actively in oral health practices. In contrast, the interaction between gender and age was not statistically significant for knowledge or attitude, although a slight upward trend in attitude was observed among women aged 31‐40 years (interaction coefficient=1.50; *P*=.06). The interaction between educational attainment and annual income showed a predominantly negative association with attitude scores, particularly among higher-educated individuals within the same income bracket. Notably, individuals with high income and a bachelor’s degree exhibited significantly higher attitude scores compared to other groups (interaction coefficient=3.13; *P*<.05). No significant differences were observed in knowledge or practice dimensions.

## Discussion

### Principal Findings

Drawing on a convenience sample from Northeast China, this study uses descriptive statistics and multivariate regression analyses, including interaction terms, to examine the relationships between sociodemographic variables and periodontal KAP scores. The study’s novelty lies in addressing the absence of KAP research on periodontitis in Northeast China and in uncovering nuanced sociodemographic influences through interaction regression analysis. By extending the analytical framework beyond descriptive statistics, this research offers generalizable methodologies and insights that can guide oral health policy development and educational interventions in other regions experiencing similar disparities. Furthermore, this study included adolescents aged 12‐17 years and incorporated gingival bleeding as an early marker of periodontal disease, thereby enhancing the comprehensiveness of population-level assessment.

Most participants lacked sufficient awareness of periodontitis and effective measures to maintain periodontal health. This suggests that awareness and oral care practices related to periodontitis in Northeast China have not significantly improved over the past decade, indicating persistent inadequacies in public health policies related to periodontal health. The findings revealed poor overall knowledge and practices, consistent with previous studies. While the findings of this study bear some similarity to previous research [19,21], a key distinction lies in the inclusion of the age group 12‐17 years for the first time. Results indicate that this age group had significantly higher practice scores and a higher detection rate of gingival bleeding; however, markedly lower scores in knowledge and attitude. This may be attributed to parental supervision, which promotes daily periodontal maintenance and thereby elevates practice scores in this group [22,23]. However, such externally imposed routines may not foster genuine improvements in knowledge or attitudes toward periodontal disease. Thus, motivational oral health education may be more effective in enhancing adolescents’ understanding of periodontal disease.

However, it is encouraging that attitudes toward periodontitis have shown significant improvement, which may be attributed to several factors. First, there appears to be a shift in public perception of the disease. Recent research has highlighted the association between periodontitis and various systemic diseases [24], demonstrating that it is not merely an isolated oral condition. This connection may have heightened public concern, thereby improving attitudes toward the disease. Second, the influence of social media should not be overlooked. Social media platforms have played a key role in increasing public awareness of oral health [25]. Although much of the evidence supporting this comes from international studies, the impact of media on raising health awareness is undeniable. Finally, the rapid aging of China’s population over the past 40 years [26] has also contributed to heightened awareness. The prevalence and severity of periodontitis increase with age [27], leading to higher dental consultation rates among older populations. In addition, interventions by health care professionals have had a positive effect on improving attitudes toward periodontal health [28].

In recent decades, China’s economic growth [29] and significant improvements in education levels have likely contributed to improved attitudes toward periodontitis. This study found that increased income and education levels positively correlated with higher KAP scores regarding periodontitis. This finding aligns with previous research but places particular emphasis on older male populations [30]. In addition, studies have shown that respondents in lower income categories are approximately 3 times more likely to report poor self-reported oral health compared to those in the highest income categories. Similarly, individuals with less than a high school education are about 1.5 times more likely to report poor self-reported oral health compared to those with higher education levels [31].

Age had a notable impact on KAP scores, with stronger correlations observed in younger populations. Among older individuals, the relationship was less pronounced, likely due to cognitive decline associated with aging, increased difficulty in acquiring new knowledge, and limited access to periodontal health information and resources [32]. Interestingly, gender demonstrated an age-dependent effect on KAP scores. While gender alone did not show a significant impact on KAP, females consistently outperformed males in practice. Furthermore, this trend rose with increasing age. Gender differences are often overlooked in clinical practice [33], underscoring the need to prioritize gender-based considerations in the clinical diagnosis and treatment of periodontitis. Oral health education campaigns should also address gender differences to deliver more targeted and effective interventions.

This study also found that individuals with gingival bleeding exhibited slightly better overall KAP scores, consistent with previous research [20]. Gingival bleeding is an early symptom of gingivitis [34]. Although self-reported gingival bleeding on brushing has low sensitivity for diagnosing periodontitis, it serves as an important sentinel sign of gingival inflammation and is visually detectable even with minimal blood loss [35]. Consequently, timely dental visits following the detection of gingival bleeding, coupled with health education and professional intervention, are associated with improved KAP scores [28]. However, disappointingly, a cross-sectional study on periodontal health KAP among Chinese adults reported that only 6.4% of individuals with gingival bleeding sought professional dental care [19]. Most people choose not to seek professional help even after noticing symptoms, which may be attributed to low oral health awareness, dental anxiety, or other related factors [36].

Studies have shown that oral health education and promotional interventions can significantly increase patients’ willingness to seek dental care and positively influence their attitudes and practices [37]. Community-based health education programs have been identified as effective strategies to raise public awareness of oral health and improve periodontal KAP [38,39], especially in enhancing the oral health knowledge of children and mothers [39]. Therefore, community-based oral health prevention institutions should strengthen efforts to promote oral health education and training, teaching the public how to respond appropriately to gingival bleeding and ultimately increasing dental visit rates.

### Limitations

This study has several limitations. First, there was some bias in the representativeness of the sample, as sample sizes were not evenly distributed across all groups. However, significance testing was performed for all categories to ensure the statistical reliability of the data. Second, all collected data were self-reported by participants, which may have introduced potential biases, including social desirability and recall biases. As a result, scores for attitudes and practices might have been affected. Third, the use of convenience sampling likely introduced selection bias, since participants were recruited based on availability and willingness rather than through a fully randomized process. Fourth, participants’ voluntary enrollment in the survey created a self-selection bias: those who chose to respond may differ systematically in their periodontal knowledge, attitudes, or practices from those who did not participate. Nevertheless, the large sample size allowed the study to capture specific characteristics and distributions of periodontal KAP in Northeast China.

### Conclusion

In conclusion, this study used a large, community-representative sample to assess KAP concerning periodontitis among populations in Northeast China. The KAP concerning periodontitis in Northeast China has improved to some extent but remains relatively low overall. Furthermore, regional, income, educational, and gender differences in KAP regarding periodontitis demonstrate complex interactions, highlighting the need for additional evaluative factors in future research. Gingival bleeding significantly influences KAP and underscores the critical role of oral health care professionals in enhancing public awareness of periodontitis through clinical education programs.

Therefore, policy interventions should prioritize community-based oral health education, particularly in economically disadvantaged and rural regions. Such programs should focus on high-risk populations and leverage early indicators, such as gingival bleeding, to prompt timely professional consultation. Efforts to enhance oral hygiene practices and motivation among older men may also contribute to improving the population’s overall KAP regarding oral health. Addressing attitude-related misalignments among highly educated individuals is also a pressing concern. Moreover, policies aimed at minors should emphasize motivational rather than mandatory oral health education, empowering parents to actively support children’s understanding and adoption of periodontal care practices. Integrating oral health education into school curricula and workplace wellness programs could further reinforce preventive behaviors from an early stage. Future research should explore underlying determinants to more effectively advance population-level oral health literacy and behavior.

## Abbreviations

KAP: Knowledge, Attitudes, and Practices
